# Supplementary material for: Determinants of self-reported health status during COVID-19 lockdown among surveyed Ecuadorian population: A cross sectional study
Source: PLoS One. 2023 Mar 8;18(3):e0275698. doi: 10.1371/journal.pone.0275698 (PMC9994680; doi:10.1371/journal.pone.0275698)
Supplement: S1 File — (DOCX) [file pone.0275698.s002.docx]

**S1 File. - Spanish version of the “Survey on the social impact of confinement / quarantine / social isolation due to the coronavirus covid-19 outbreak in Latin America” carried out to assess the social impact of COVID-19 lockdown in Ecuador.**

**ENCUESTA SOBRE EL IMPACTO SOCIAL DEL CONFINAMIENTO / CUARENTENA / AISLAMIENTO SOCIAL POR EL BROTE DE CORONAVIRUS COVID-19 EN LATINOAMERICA**

Le invitamos a participar en esta encuesta para conocer cómo está viviendo la situación de confinamiento / cuarentena / aislamiento social, y el impacto social de la pandemia. El objetivo del estudio es evaluar y comparar el impacto social durante y después del período de confinamiento aplicado como medida de prevención y control del SARS-CoV-2 en la población mayor de 18 años residente en Brasil, Chile, Ecuador, España y México. Con esta información podremos planificar acciones para ayudar a la población y mejorar los protocolos en futuras pandemias.

**¿Quiénes somos?**

Somos un grupo de investigador@s de FIOCRUZ Brasilia, **Brasil,** de la Escuela de Salud Pública de la Universidad de **Chile,** del Instituto de Salud Pública de la Universidad Católica del **Ecuador**, del Institut Universitari d'Atenció Primària IDIAPJGol (**España**) y del Instituto Nacional de Salud Pública México, Escuela de Salud Pública de **México**.

**¿Quiénes pueden participar?**

En esta encuesta pueden participar personas que viven en Ecuador que sean mayores de 18 años. Su participación es totalmente voluntaria y puede retirarse o dejar de contestar en el momento que así lo desee.

**¿Cuánto tiempo le llevará llenar la encuesta?**

Le llevará unos 10 a 15 minutos aproximadamente.

**¿Mis datos están protegidos?**

Sí, esta plataforma que utilizamos para realizar la encuesta on-line cumple con todos los criterios internacionales de protección de datos. El protocolo de estudio tiene las aprobaciones éticas requeridas bajo la normativa actual de cada uno de los países participantes.

**Contacto**

Si requiere mayor información, se puede contactar por correo electrónico con Ana Lucía Torres ([ATORRES331@puce.edu.ec](about:blank)) o Andrés Peralta ([tirico85@gmail.com](about:blank)) o por vía telefónica con el número del Instituto de Salud Pública de la PUCE (593 2 299 1684).

**□ Acepto participar en la investigación. He leído y comprendido en que consiste mi participación.**

**Nota:** A lo largo de esta encuesta, usamos el término confinamiento para describir la situación de cuarentena y/o medidas de aislamiento social (limitación movilidad, cierre trabajos, universidades, escuelas etc.) implementadas en cada país.

**Datos sociodemográficos**

| 1. **¿En qué país está viviendo el confinamiento?** |  |
| --- | --- |

**(Desplegable con opciones: Ecuador)**

| 1. **¿En qué provincia está viviendo el confinamiento?** |  |
| --- | --- |

**(Desplegable con 24 provincias del Ecuador)**

| 1. **¿En qué cantón y parroquia está viviendo el confinamiento?** | **Cantón:**  **Parroquia:** |
| --- | --- |

**(Preguntas abiertas)**

| 1. **Sexo** |
| --- |
| Hombre |
| Mujer |
| Otro: Especificar _____________________________ |

| 1. **¿Qué edad tiene?** | **_____años** |
| --- | --- |

| 1. **¿Cómo se autoidentifica según su cultura y costumbres?** |
| --- |
| Indígena |
| Afroecuatoriano / Afrodescendiente |
| Negro/a |
| Mulato/a |
| Montubio/a |
| Mestizo/a |
| Blanco/a |
| Otro: Especificar _____________________________ |

| 1. **¿Cuál es su país de nacimiento?** |
| --- |

**(Desplegable con países)**

| 1. **¿Cuál es su situación migratoria actualmente?** |
| --- |
| Tengo nacionalidad ecuatoriana |
| Residencia temporal o permanente |
| Sin papeles o en trámites |

**(Aparece si país de nacimiento no coincide con país de confinamiento)**

| 1. **¿Cuál es su nivel máximo de estudios finalizados?** |
| --- |
| Sin escolarización oficial |
| Primaria |
| Educación básica |
| Educación media / Bachillerato |
| Educación técnica o tecnológica |
| Educación universitaria o superior (por ejemplo, licenciatura, maestría etc.) |
| Otro 🡪 especifique ____________ |
| No sabe |

| 1. **¿Cuál era su situación laboral antes del confinamiento?** |
| --- |
| Empleado/a del sector público |
| Empleado/a del sector privado |
| Trabajo por cuenta propia |
| Trabajo sin remuneración |
| Estudiante |
| Trabajos domésticos no remunerados (Ama de casa) |
| Jubilado/a |
| Otra 🡪 especifique ____________ |

| 1. **¿Usted tiene acceso a alguno de los siguientes servicios de salud? (marque el principal):** |
| --- |
| Instituto Ecuatoriano de Seguridad Social (IESS) |
| Otros servicios de seguros públicos (ISSFA / ISSPOL) |
| Seguro médico privado |
| Ministerio de Salud Pública (MSP) |
| Otro 🡪 especifique ____________ |

**Contexto durante el confinamiento**

| 1. **¿Cuántos días lleva en confinamiento?** | **________ días**  **□ No estoy en confinamiento** |
| --- | --- |

| 1. **¿Cuál es su situación actual en relación al confinamiento? (puede seleccionar más de una opción)** |
| --- |
| Estoy realizando confinamiento obligado (por políticas de gobierno) |
| Estoy realizando confinamiento voluntario |
| Estoy realizando confinamiento pero me dedico a trabajos esenciales (salud, transporte público, alimentación,…) |
| No estoy realizando confinamiento porque económicamente no puedo |
| No estoy realizando confinamiento porque mi empresa no me deja teletrabajar |
| No estoy realizando confinamiento porque no creo en las medidas del gobierno |
| No estoy realizando confinamiento porque creo que el coronavirus no me afecta |
| No estoy realizando confinamiento porque creo que el coronavirus no es tan grave |
| Otra🡪 especifique ____________ |

| 1. **¿Qué trabajo esencial realiza?** |
| --- |
| Relacionado a la alimentación tanto de personas o de animales |
| Relacionado a la salud |
| Relacionado a las fuerzas y cuerpos de seguridad del Estado (militares / policías) |
| Relacionado a medios de comunicación |
| Relacionado al transporte público |
| Relacionado con personal de limpieza |
| Relacionado con recolección de basura |
| Relacionado a la atención de colectivos o personas vulnerables (cuidados físicos o psicosociales) |
| Otro🡪 especifique ____________ |
|  |

| 1. **Si usted realiza alguna actividad esencial durante el confinamiento,** **¿cree que cuenta con el equipamiento adecuado para protegerse del coronavirus?** | Sí | No | No sé |
| --- | --- | --- | --- |

**(Aparece sólo si realiza alguna actividad esencial, respuesta si en la 14)**

| 1. **Si usted realiza alguna actividad esencial durante el confinamiento, ¿ha sufrido insultos o agresiones por estar trabajando durante la pandemia del coronavirus?** | Sí | No | No sé |
| --- | --- | --- | --- |

**(Aparece sólo si realiza alguna actividad esencial, respuesta si en la 14)**

| 1. **¿El confinamiento ha afectado de alguna manera a su situación laboral?** |
| --- |
| No, no ha cambiado |
| Sí, ha empeorado un poco |
| Sí, ha empeorado mucho |
| Sí, pero no ha mejorado ni empeorado el |
| Sí, ha mejorado un poco |
| Sí, ha mejorado mucho |

1. **¿Cuántas veces sale de casa a la semana?**

**(Opciones fijas entre 0 y 14 o más)**

| 1. **¿Por qué motivo ha salido de casa? (Puede seleccionar más de una opción)** |
| --- |
| Pasear al perro |
| Salir a comprar |
| Salir a cuidar personas (adultos mayores, niñ@s, personas enfermas o con discapacidad) |
| Salir a trabajar |
| Salir a dar un paseo o hacer deporte |
| Salir para ver a la familia o amigo/as |
| Otro🡪 especifique ____________ |

| 1. **¿Con cuántas personas vive durante el confinamiento?** (Incluyéndole a usted) |  |
| --- | --- |

**(Opciones fijas entre 1 y 10 o más)**

| 1. **¿Con cuántas personas menores de 18 años vive el confinamiento?** |  |
| --- | --- |

**(Opciones fijas entre 1 y 9 o más)**

| 1. **¿Con cuántas personas que requieren cuidados por su edad, salud o situación de discapacidad vive el confinamiento?** |  |
| --- | --- |

**(Opciones fijas entre 1 y 9 o más)**

| 1. **Su vivienda es:** |
| --- |
| Propia (totalmente pagada) |
| Propia (pagando crédito hipotecario) |
| Arrendada |
| Arriendo de una sola habitación |
| Otra situación 🡪 especifique __________________________ |

| 1. **¿Cuántos metros cuadrados aproximados tiene la vivienda donde se encuentra confinado?** | | | | |
| --- | --- | --- | --- | --- |
| Menos de 50 m^2^ | De 50 a 80 m^2^ | De 80 a 100 m^2^ | De 100 a 120 m^2^ | Más de 120 m^2^ |

| 1. **¿La vivienda donde se encuentra confinado tiene alguna de las siguientes características?** |
| --- |
| Balcón o terraza |
| Jardín privado |
| Otras zonas exteriores |
| Ningún espacio exterior |

| 1. **¿Cree que su vivienda es adecuada para afrontar el confinamiento? (espacio, luz, número de habitaciones, etc.)** | | | | |
| --- | --- | --- | --- | --- |
| Nada | Un poco | Moderadamente | Bastante | Mucho |

| 1. **¿En qué medida le preocupa la convivencia con los miembros de su hogar durante el confinamiento?** | | | | |
| --- | --- | --- | --- | --- |
| Nada preocupad/a | Un poco preocupado/a | Moderadamente preocupado/a | Bastante preocupado/a | Muy preocupado/a |

| 1. **En caso de que tenga menores a cargo ¿cuánto le preocupa su formación escolar durante el confinamiento?** | | | | |
| --- | --- | --- | --- | --- |
| Nada preocupado/a | Un poco preocupado/a | Moderadamente preocupado/a | Bastante preocupado/a | Muy preocupado/a |

| 1. **La carga del trabajo doméstico y/o de cuidados de niños/as y/o adultos dependientes en el hogar se distribuye:** | | | | |
| --- | --- | --- | --- | --- |
| Equitativamente entre todos los miembros | La realizo mayoritariamente yo | La realizan mayoritariamente otras personas | Otra, especifica_________ |  |

| 1. **¿Ha sufrido algún tipo de violencia (o maltrato) en el hogar durante el confinamiento?** | | | | |
| --- | --- | --- | --- | --- |
| Nada | Un poco | Moderadamente | Bastante | Mucho |

| 1. **Especifique el tipo de violencia ha padecido y conviviente (puede contestar más de una opción)** | **Pareja** | **Madre, Padre, Cuidador** | **Hijo/a** | **Otra persona del hogar** |
| --- | --- | --- | --- | --- |
| Violencia verbal o psicológica |  |  |  |  |
| Violencia física |  |  |  |  |
| Violencia sexual |  |  |  |  |
| Violencia económica |  |  |  |  |

| 1. **A nivel general, percibe que en su hogar la violencia durante el confinamiento…** |
| --- |
| Ha aumentado |
| Ha disminuido |
| Es la misma |
| No sé |

**Salud**

| 1. **¿Cómo diría usted que es su salud en general?** |
| --- |
| Excelente |
| Muy buena |
| Buena |
| Regular |
| Mala |
| No sé |

| 1. **¿Tiene alguna enfermedad o problema de salud crónico o de larga duración? Entendemos por larga duración si el problema de salud ha durado o se espera que dure 6 meses o más.** |
| --- |
| Si |
| No |
| No sé |

| 1. **¿Usted está o ha estado diagnosticado/a por coronavirus?** (Diagnosticado / informado por un profesional de la salud) | Sí | No |
| --- | --- | --- |

| 1. **¿Usted tiene o ha tenido síntomas como tos, fiebre, dificultad para respirar y/u otros síntomas compatibles con el coronavirus?** | Sí | No |
| --- | --- | --- |

| 1. **¿Le han realizado prueba(s) diagnóstica(s) para confirmar el coronavirus?** |
| --- |
| Si, me realizaron al menos una prueba para confirmar el diagnóstico y me entregaron el resultado. |
| Si, me tomaron muestras para la prueba, pero no me han confirmado el resultado. |
| No, no me realizaron la prueba por falta de pruebas o reactivos |
| No, no me realizaron la prueba por que mi condición no era lo suficientemente grave |
| No sé |
| Otro🡪 especifique __________________________ |

**(Aparece sólo si ha sido diagnosticado o tiene síntomas por coronavirus, si responde si 32 o 33)**

| 1. **¿Ha tenido miedo o vergüenza de explicar que tiene coronavirus (o síntomas) por si le perciben o tratan de forma negativa?** | Sí | No |
| --- | --- | --- |

**(Aparece sólo si ha sido diagnosticado o tiene síntomas)**

| 1. **¿Ha sufrido insultos o agresiones por estar diagnosticado/a o con síntomas de coronavirus?** | Sí | No |
| --- | --- | --- |

**(Aparece sólo si ha sido diagnosticado o tiene síntomas por coronavirus, si responde si 32 o 33)**

| 1. **¿Alguna persona con la que convive está o ha estado diagnosticado de coronavirus?** (Diagnosticado/informado por un profesional de la salud) | Sí | No |
| --- | --- | --- |

| 1. **¿Alguna persona con la que convive tiene o ha tenido síntomas como tos, fiebre, dificultad de respirar y/u otros síntomas compatibles con el coronavirus?** | Sí | No |
| --- | --- | --- |

| 1. **¿Alguna persona cercana ha fallecido durante el confinamiento?** | Sí | No |
| --- | --- | --- |

| 1. **¿Cree que esta persona ha fallecido por coronavirus?** | Sí | No | No sé |
| --- | --- | --- | --- |

**(Aparece sólo si alguna persona cercana a fallecido)**

| 1. **Actualmente ¿en qué medida otras personas de su entorno (familia, amigos, etc.) se interesan por lo que a usted le pasa o hace?** |
| --- |
| Ningún interés |
| Poco interés |
| Interés incierto |
| Mucho interés |
| No sé |

| 1. **Actualmente ¿en qué medida le resultaría fácil obtener ayuda de los vecinos en caso de necesidad?** |
| --- |
| Muy difícil |
| Difícil |
| Posible |
| Fácil |
| Muy fácil |
| No sé |

| 1. **Durante el confinamiento, ¿ha recibido alguna ayuda económica o social por parte del gobierno central, provincial o municipal?** | Sí | No | No sé |
| --- | --- | --- | --- |

| 1. **Si la respuesta es afirmativa ¿Especifique el tipo de ayuda recibida?** |
| --- |
| Ayuda económica (bono) |
| Alimentos y productos para el hogar |
| Otro🡪 especifique __________________________ |

**(Aparece sólo si ha sido ha recibido alguna ayuda, si responde si en la 44)**

| 1. **Durante el confinamiento, ¿Está realizando alguna actividad nueva (meditación, yoga, actividades asociativas, etc.)?** | Sí | No | No sé |
| --- | --- | --- | --- |

| 1. **, ¿considera que esta nueva actividad es beneficiosa para su salud?** | Sí | No | No sé |
| --- | --- | --- | --- |

**Percepción del riesgo**

| 1. **¿Qué grado de preocupación tiene de infectarse por coronavirus?** | | | | |
| --- | --- | --- | --- | --- |
| Nada preocupado | Un poco preocupado | Moderadamente preocupado | Bastante preocupado | Muy preocupado |

| 1. **¿Qué grado de preocupación tiene de que algún familiar o amigo/a suyo se contagie por coronavirus?** | | | | |
| --- | --- | --- | --- | --- |
| Nada preocupado | Un poco preocupado | Moderadamente preocupado | Bastante preocupado | Muy preocupado |

| 1. **¿Considera que el coronavirus es grave problema para la salud de la población?** | | | | |
| --- | --- | --- | --- | --- |
| Totalmente en desacuerdo | Un poco en desacuerdo | Ni de acuerdo, ni en desacuerdo | Un poco de acuerdo | Totalmente de acuerdo |

| 1. **¿Considera que el coronavirus es un grave problema para su economía?** | | | | |
| --- | --- | --- | --- | --- |
| Totalmente en desacuerdo | Un poco en desacuerdo | Ni de acuerdo, ni en desacuerdo | Un poco de acuerdo | Totalmente de acuerdo |

| 1. **¿Considera que ha tenido suficiente información sobre las medidas de prevención del coronavirus?** | | | | |
| --- | --- | --- | --- | --- |
| Totalmente en desacuerdo | Un poco en desacuerdo | Ni de acuerdo, ni en desacuerdo | Un poco de acuerdo | Totalmente de acuerdo |

| 1. **¿Considera que las medidas implementadas por el gobierno para el control del coronavirus han sido las adecuadas?** | | | | |
| --- | --- | --- | --- | --- |
| Totalmente en desacuerdo | Un poco en desacuerdo | Ni de acuerdo, ni en desacuerdo | Un poco de acuerdo | Totalmente de acuerdo |

| 1. **¿Cree que el gobierno debió haber implementado el confinamiento antes?** |
| --- |
| Si y lo hubiera cumplido |
| Si, pero no lo habría cumplido |
| No, creo que fue en el momento justo |
| No, creo que hubiera sido mejor después |
| Creo que no era necesario haber asumido la medida de confinamiento |

**COMPORTAMIENTO Y SALUD EMOCIONAL**

| 1. **Durante el confinamiento, ¿Usted ha cambiado estos comportamientos?** | | | | |
| --- | --- | --- | --- | --- |
| **Consumo de tabaco** | No consumo | He aumentado el consumo | He mantenido el mismo consumo | He disminuido el consumo |
| **Consumo de alcohol** | No consumo | He aumentado el consumo | He mantenido el mismo consumo | He disminuido el consumo |
| **Consumo de pastillas (ansiolíticos)** | No consumo | He aumentado el consumo | He mantenido el mismo consumo | He disminuido el consumo |
| **Consumo de drogas no legales (marihuana, cocaína)** | No consumo | He aumentado el consumo | He mantenido el mismo consumo | He disminuido el consumo |
| **Consumo de comida rápida (hamburguesas, pizzas, etc.)** | No consumo | He aumentado el consumo | He mantenido el mismo consumo | He disminuido el consumo |
| **Consumo de bebidas azucaradas** | No consumo | He aumentado el consumo | He mantenido el mismo consumo | He disminuido el consumo |
| **Niveles de actividad física** | No realizo | He aumentado el nivel | He mantenido el mismo nivel | He disminuido el nivel |
| **Consumo de televisión o plataformas digitales (Netflix, HBO, etc.)** | No consumo | He aumentado el consumo | He mantenido el mismo consumo | He disminuido el consumo |
| **Consumo de redes sociales (Instagram, Facebook, Twitter, etc.)** | No consumo | He aumentado el consumo | He mantenido el mismo consumo | He disminuido el consumo |

| 1. **Durante las últimas 2 semanas, ¿qué tan seguido ha tenido molestias debido a los siguientes problemas?** | **Nunca** | **Menos de la mitad de los días** | **Más de la mitad de los días** | **Casi todos los días** |
| --- | --- | --- | --- | --- |
| Se ha sentido nervioso, ansioso o muy alterado |  |  |  |  |
| No ha podido dejar de preocuparse |  |  |  |  |
| Se ha preocupado excesivamente por diferentes cosas |  |  |  |  |
| Ha tenido dificultad para relajarse |  |  |  |  |
| Se ha sentido tan intranquilo que no podía estarse quieto |  |  |  |  |
| Se ha irritado o enfadado con facilidad |  |  |  |  |
| Ha sentido miedo, como si fuera a suceder algo terrible |  |  |  |  |

| 1. **Durante las últimas 2 semanas, ¿qué tan seguido ha tenido molestias debido a los siguientes problemas?** | **Nunca** | **Menos de la mitad de los días** | **Más de la mitad de los días** | **Casi todos los días** |
| --- | --- | --- | --- | --- |
| Poco interés o placer en hacer cosas |  |  |  |  |
| Se ha sentido decaído(a), deprimido(a) o sin esperanzas |  |  |  |  |
| Dificultad en caer o permanecer dormido(a), o dormir demasiado |  |  |  |  |
| Sintiéndose cansado o teniendo poca energía |  |  |  |  |
| Poco apetito o comer en exceso |  |  |  |  |
| Sintiéndose mal con usted mismo(a) – o que usted es un fracaso o que ha quedado mal con usted mismo(a) o con su familia |  |  |  |  |
| Dificultad en concentrarse en cosas, tales como leer el periódico o ver televisión |  |  |  |  |
| ¿Moviéndose o hablando tan lento, que otras personas podrían notarlo? O lo contrario – muy inquieto(a) o agitado(a) que usted ha estado moviéndose mucho más de lo normal |  |  |  |  |
| Pensamientos de que usted estaría mejor muerto(a) o de alguna manera lastimándose a usted mismo(a) |  |  |  |  |
| Si usted marcó cualquiera de los problemas, **¿Le ha sido difícil sobrellevar su trabajo, encargarse de tareas del hogar, o llevarse bien con otras personas debido a los problemas mencionados?** | Para nada difícil | Un poco difícil | Muy difícil | Extremadamente difícil |

**Para terminar**

Opcionalmente puede responder las siguientes preguntas:

| 1. ¿Desea contar su experiencia en una entrevista telefónica? Indíquenos su número de teléfono o email para que le podamos contactar. |  |
| --- | --- |

| 1. ¿Estaría dispuesto/a a contestar una encuesta de seguimiento en 6 meses? Déjenos su email. |  |
| --- | --- |

**¡MUCHAS GRACIAS POR SU PARTICIPACIÓN!**

Contacto en caso de violencia de género:

- **Teléfono**: 911 o 1800-DELITO(335486) opción 4.
- **App 911**: Una vez descargada se puede pulsar el ícono "violencia intrafamiliar" en una situación de emergencia.
- **Organizaciones y Colectivos de Mujeres**:
  - CEPAM Guayaquil - 0991113526
  - SURKUNA - 0999928032
  - Warmi Pichincha – 0987427448

Teléfonos de asistencia psicológica por Coronavirus:

- Centro de Psicología Aplicada PUCE – (02) 299 1641
- Atención Psicológica Municipio de Quito - 099 349 69
