# Supplementary material for: Determinants of self-reported health status during COVID-19 lockdown among surveyed Ecuadorian population: A cross sectional study
Source: PLoS One. 2023 Mar 8;18(3):e0275698. doi: 10.1371/journal.pone.0275698 (PMC9994680; doi:10.1371/journal.pone.0275698)
Supplement: S1 Table — (DOCX) [file pone.0275698.s003.docx]

*S1Table. Description of the sample and missing values per each variable.*

| ***Variable*** | ***Whole sample***  ***n= 2924*** | ***Number of missing values***  ***n (%)*** |
| --- | --- | --- |
| Age in years of life, median (IQR) | 34 (27 to 44) | 261 (8.9) |
| Female, n % | 1801 (68) | 269 (9.2) |
| Education level |  |  |
| *Educational level lower than university education, n %* | 408 (16) | 325 (11.1) |
| *University educational level or higher, n %* | 2191 (84) | - |
| Employment status |  |  |
| *Public or private full job, n (%)* | 1601 (63) | 396 (13.5) |
| *Self-employment, n (%)* | 315 (13) | - |
| *Unpaid work, retired or student, n (%)* | 612 (24) | - |
| Access to health services |  | 434 (14.8) |
| *Social security^a^, n (%)* | 1517 (61) | - |
| *Private health insurance, n (%)* | 598 (24) | - |
| *Public health services user, n (%)* | 375 (15) | - |
| Perception of the adequacy of the type of housing to lockdown |  |  |
| *Moderately to well adequate, n (%)* | 2204 (86) | 372 (12.7) |
| *Little or not adequate, n (%)* | 348 (14) | - |
| Housing area |  |  |
| *<50 m^2^, n (%)* | 243 (10) | 383 (13.1) |
| *50 to 80 m^2^, n (%)* | 488 (19) | - |
| *80 to 100 m^2^, n (%)* | 557 (22) | - |
| *100 to 120 m^2^, n (%)* | 477 (19) | - |
| *≥120 m^2^, n (%)* | 779 (31) | - |
| Number of cohabitants, median (IQR) | 4 (2 to 5) | 363 (12.4) |
| Number of cohabitants who require care, median (IQR) | 2 (1 to 3) | 644 (22.0) |
| Number of cohabitants <18 years old, median (IQR) | 2 (1 to 3) | 631 (21.6) |
| Physical activity during lockdown |  |  |
| *Not performing, n (%)* | 366 (16) | 607 (20.8) |
| *Increased performing, n (%)* | 588 (25) | - |
| *The same performing than before lockdown, n (%)* | 492 (21) | - |
| *Reduced performing, n (%)* | 871 (38) | - |
| Alcohol consumption |  |  |
| *Increase of alcohol consumption during lockdown, n (%)* | 111 (5) | 605 (20.7) |
| *Any alcohol consumption during lockdown, n (%)* | 825 (36) | 605 (20.7) |
| Any cigarette consumption during lockdown, n (%) | 229 (10) | 606 (20.7) |
| Any illicit drugs consumption during lockdown, n (%) | 83 (4) | 600 (20.5) |
| Any consumption of sugary drinks, n (%) | 1593 (67) | 604 (20.7) |
| Concerns arising from the pandemic: degree of concern of being infected with SARS-CoV-2 |  |  |
| *Not worried, n (%)* | 69 (3) | 592 (20.2) |
| *A little worried, n (%)* | 295 (13) | - |
| *Moderately worried, n (%)* | 781 (33) | - |
| *Quite worried, n (%)* | 654 (28) | - |
| *Very worried, n (%)* | 533 (23) | - |
| Very high difficulties to cope with the job or take care of household chores, n (%) | 109 (5) | 613 (21.0) |
| New health activities during lockdown, n (%) | 1187 (51) | 606 (20.7) |
| Suffer any type of violence or abuse during lockdown, n (%) | 316 (14) | 648 (22.2) |
| Diseases, symptoms, and medications |  |  |
| Have or had COVID-19 | 266 (11) | 411 (14.1) |
| Presence of any chronic disease | 789 (34) | 566 (19.4) |
| Anxiety symptoms as measured by GAD-7 questionnaire |  | 621 (21.2) |
| *No anxiety (<5 points), n (%)* | 539 (23) | - |
| *Mild anxiety (5 to <10 points), n (%)* | 801 (35) | - |
| *Moderate anxiety (10 to <15 points), n (%)* | 579 (25) | - |
| *Severe anxiety (≥15 points), n (%)* | 384 (17) | - |
| *Any anxiety level (≥5 points), n (%)* | 1740 (76) | - |
| Depression symptoms as measured by PHQ9 questionnaire |  |  |
| *No depression (<5 points), n (%)* | 708 (31) | 626 (21.4) |
| *Mild depression (5 to <10 points), n (%)* | 700 (30) | - |
| *Moderate depression (10 to <15 points), n (%)* | 473 (21) | - |
| *Moderately severe depression (15 to <20 points), n (%)* | 256 (11) | - |
| *Severe depression (≥20 points), n (%)* | 161 (7) | - |
| *Any depression level (≥5 points), n (%)* | 1590 (69) | - |
| Any use of antidepressants, n (%) | 225 (10) | 602 (20.6) |
| Poor or regular health self-perception | 386 (16) | 427 (14.6) |
| IQR = Interquartile range  GAD-7 = Generalized Anxiety Disorder Scale  PHQ9 = Patient Health Questionnaire  *^a^* = It corresponds to the beneficiaries of the Ecuadorian Institute of Social Security (IESS, for its acronym in Spanish), the social security of the armed forces (ISSFA, for its acronym in Spanish) and the social security of the police (ISSPOL, for its acronym in Spanish). acronym in Spanish) | | |
