# Supplementary material for: Determinants of self-reported health status during COVID-19 lockdown among surveyed Ecuadorian population: A cross sectional study
Source: PLoS One. 2023 Mar 8;18(3):e0275698. doi: 10.1371/journal.pone.0275698 (PMC9994680; doi:10.1371/journal.pone.0275698)
Supplement: S2 Table — (DOCX) [file pone.0275698.s004.docx]

*S2 Table. - Adjusted Odds Ratios of regular or bad health self-perception excluding: (i) high and low educated subjects, (ii) those with chronic diseases, (iii) those with severe anxiety, and (iv) those with severe depression.*

| ***Variable*** | ***Excluding highly educated subjects***  ***n=408*** | ***p-value*** | ***Excluding less educated subjects***  ***n=2191*** | ***p-value*** | ***Excluding subjects with any chronic disease***  ***n=789*** | ***p-value*** | ***Excluding subjects with severe anxiety***  ***n=1919*** | ***p-value*** | ***Excluding subjects with severe depression***  ***n=2137*** | ***p-value*** |
| --- | --- | --- | --- | --- | --- | --- | --- | --- | --- | --- |
| Female *(male is the ref.)* | *2.4 (0.9 to 6.4)* | *0.073* | *1.5 (1.0 to 2.2)* | *0.060* | *1.1 (0.6 to 2.1)* | *0.681* | *1.6 (1.0 to 2.4)* | *0.029* | *1.6 (1. 1 to 2.4)* | *0.017* |
| Employment status |  |  |  |  |  |  |  |  |  |  |
| *Public or private full job (ref.)* | *1* | *-* | *1* | *-* | *1* | *-* | *1* | *-* | *1* | *-* |
| *Self-employment* | *1.0 (0.2 to 4.9)* | *0.961* | *0.4 (0.2 to 0.8)* | *0.005* | *0.3 (0.1 to 1.3)* | *0.104* | *0.4 (0.2 to 0.9)* | *0.019* | *0.5 (0.3 to 0.9)* | *0.033* |
| *Unpaid work, retired or student* | *0.9 (03 to 2.9)* | *0.912* | *0.7 (0.4 to 1.2)* | *0.208* | *1.2 (0.6 to 2.3)* | *0.647* | *0.9 (0.5 to 1.4)* | *0.527* | *1.0 (0.7 to 1.6)* | *0.893* |
| Access to health services |  |  |  |  |  |  |  |  |  |  |
| *Social security^a^ (ref.)* | *1* | *-* | *1* | *-* | *1* | *-* | *1* | *-* | *1* | *-* |
| *Private health insurance* | *0.7 (0.2 to 2.1)* | *0.469* | *0.6 (0.4 to 1.1)* | *0.080* | *0.5 (0.2 to 1.1)* | *0.077* | *0.9 (0.6 to 1.6)* | *0.841* | *0.8 (0.5 to 1.2)* | *0.258* |
| *Public health services user* | *1.4 (0.5 to 4.1)* | *0.557* | *2.0 (1.2 to 3.3)* | *0.013* | *1.2 (0.6 to 2.5)* | *0.578* | *2.0 (1.2 to 3.5)* | *0.011* | *1.7 (1.1 to 2.9)* | *0.031* |
| Perception of the adequacy of the type of housing to lockdown |  |  |  |  |  |  |  |  |  |  |
| *Little or not adequate (Moderately to well adequate is ref.)* | *1.4 (0.4 to 4.3)* | *0.573* | *2.4 (1.5 to 3.9)* | *<0.001* | *2.0 (1.1 to 3.4)* | *0.021* | *2.2 (1.3 to 3.7)* | *0.003* | *2.1 (1.3 to 3.3)* | *0.002* |
| Number of cohabitants who require care *(per each increase in one cohabitant)* | *1.3 (0.9 to 1.7)* | *0.138* | *1.2 (1.1 to 1.4)* | *0.005* | *1.1 (0.9 to 1.4)* | *0.171* | *1.3 (1.1 to 1.5)* | *0.001* | *1.2 (1.1 to 1.4)* | *0.001* |
| Very high difficulties to cope with the job or take care of household chores *(not having is the ref.)* | *2.6 (0.5 to 12.7)* | *0.226* | *2.5 (1.2 to 5.0)* | *0.010* | *2.3 (0.9 to 5.9)* | *0.091* | *2.0 (0.7 to 6.0)* | *0.198* | *2.0 (0.9 to 4.6)* | *0.090* |
| Diseases, symptoms, and medications |  |  |  |  |  |  |  |  |  |  |
| *Have or had COVID-19 (not having is the ref.)* | *1.4 (0.4 to 4.9)* | *0.603* | *3.4 (2.2 to 5.4)* | *<0.001* | *5.5 (3.0 to 10.0)* | *<0.001* | *3.4 (2.1 to 5.5)* | *<0.001* | *3.4 (2.2 to 5.3)* | *<0.001* |
| *Presence of any chronic disease (not having is the ref.)* | *8.3 (3.4 to 20.6)* | *<0.001* | *7.0 (4.8 to 10.2)* | *<0.001* | *-* | *-* | *7.1 (4.8 to 10.6)* | *<0.001* | *7.3 (5.1 to 10.6)* | *<0.001* |
| Depression symptoms as measured by PHQ-9 questionnaire, median (IQR) |  |  |  |  |  |  |  |  |  |  |
| *No depression (<5 points) (ref.)* | *1* | *-* | *1* | *-* | *1* | *-* | *1* | *-* | *1* | *-* |
| *Mild depression (5 to <10 points)* | *1.5 (0.3 to 6.3)* | *0.616* | *1.4 (0.8 to 2.4)* | *0.221* | *1.0 (0.4 to 2.5)* | *0.924* | *1.4 (0.8 to 2.3)* | *0.240* | *1.4 (0.9 to 2.4)* | *0.159* |
| *Moderate depression (10 to <15 points)* | *4.8 (1.3 to 18.4)* | *0.021* | *2.9 (1.7 to 5.0)* | *<0.001* | *3.4 (1.5 to 7.5)* | *0.003* | *2.9 (1.7 to 4.9)* | *<0.001* | *3.2 (2.0 to 5.3)* | *<0.001* |
| *Moderately severe depression (15 to <20 points)* | *8.3 (1.9 to 36.3)* | *0.005* | *3.4 (1.8 to 6.1)* | *<0.001* | *4.5 (1.9 to 10.8)* | *0.001* | *4.5 ( 2.3 to 8.5)* | *<0.001* | *3.8 (2.2 to 6.7)* | *<0.001* |
| *Severe depression (≥20 points)* | *7.0 (1.5 to 33.6)* | *0.014* | *6.4 (3.0 to 13.5)* | *<0.001* | *8.6 (3.3 to 22.9)* | *<0.001* | *3.9 (1.0 to 15.0)* | *0.05* | *-* | *-* |
| GAD-7 = Generalized Anxiety Disorder Scale  PHQ9 = Patient Health Questionnaire  *^a^* = It corresponds to the beneficiaries of the Ecuadorian Institute of Social Security (IESS, for its acronym in Spanish), the social security of the armed forces (ISSFA, for its acronym in Spanish) and the social security of the police (ISSPOL, for its acronym in Spanish). acronym in Spanish) | | | | | | | | | | |
